# Novel Threadlike Structures May Be Present on the Large Animal Organ Surface: Evidence in Swine Model

**DOI:** 10.1155/2013/758763

**Published:** 2013-05-25

**Authors:** Kyoung-Hee Bae, Sang Hyun Park, Byung-Cheon Lee, Min-Ho Nam, Ji Woong Yoon, Hee-Min Kwon, Seung Zhoo Yoon

**Affiliations:** ^1^Nano Primo Research Center, Advanced Institute of Convergence Technology, Seoul National University, Suwon 443-270, Republic of Korea; ^2^Ki Primo Research Laboratory, Division of Electrical Engineering, KAIST Institute for Information Technology Convergence, Korea Advanced Institute of Science and Technology (KAIST), Daejeon 305-701, Republic of Korea; ^3^Department of Pathology, College of Korean Medicine, Kyung Hee University, Seoul 130-701, Republic of Korea; ^4^Impedance Imaging Research Center and Department of Public Administration, Kyung Hee University, Seoul 130-701, Republic of Korea; ^5^Yonsei Institute of Convergence Technology, Yonsei University, 162-1 Sondo-dong, Yeonsu-gu, Incheon 406-840, Republic of Korea; ^6^Department of Anesthesiology and Pain Medicine, College of Medicine, Korea University, No. 5 Anam-dong Sungbuk-gu, Seoul 136-705, Republic of Korea

## Abstract

*Background*. The types of embryonic development probably provoke different paths of novel threadlike structure (NTS) development. The authors hypothesized that NTS may be easily observed on the surface of swine intestines by using trypan blue staining method and visualization under an optical microscope. *Methods*. General anesthesia was administered to 2 Yorkshire pigs. The abdominal walls of the pigs were carefully dissected along the medial alba. NTSs were identified on organ surfaces under a stereoscopic microscope after trypan blue staining. Isolated NTS specimens obtained from the large intestine were subjected to 4′,6-diamidino-2-phenylindole (DAPI) staining and observed using the polarized light microscopy to confirm whether the obtained structure fits the definition of NTS. *Results*. We found elastic, semitransparent threadlike structures (forming a network structure) that had a milky-white color *in situ *and *in vivo* in swine large intestines. The samples showed distinct extinction of polarized light at every 90 degrees, and nucleus was shown to be rod shaped by DAPI staining, indicating that they meet the criteria of NTS. *Conclusion*. We used a swine model to demonstrate that NTS may be present on large animal organ surfaces. Our results may permit similar studies by using human specimens.

## 1. Introduction

In 2003, Jiang et al. [1] authored a paper that was instrumental in establishing the existence of intravascular novel threadlike structures (NTSs). They started an intensive reinvestigation into Kim theory, which was first introduced by Kim in 1963 [2], using modernized techniques, such as fluorescent microscopy, confocal microscopy, and electron microscopy. Therefore, NTSs have been called Bonghan ducts (BDs) or primo vessels (the name used by Jiang et al.). 

Since the first discovery of NTSs, investigators have adopted various methods for confirming the existence of NTSs. These methods include confocal laser scanning microscopy; various types of electron microscopy, such as scanning electron microscopy (SEM), cryoSEM, focused-ion-beam SEM, and high voltage transmission electron microscopy (TEM); X-ray microtomography; atomic force microscopy; fluorescent nanoparticles; and immunohistochemistry. As these methods developed, NTSs were found in a gradually increasing number of organs. These organs include blood vessels of rabbits [1], lymphatic vessels of rabbits [3], lymphatic vessels of rats [4], bovine hearts [5], the central nervous system of rabbits [6], and the surfaces of rat abdominal organs (liver, stomach, and hollow viscous organs) [7, 8]. 

According to Kim [9], BDs seem to exist throughout the biological world, including in invertebrates, vertebrates, and plants. Different animal species undergo different types of embryonic development. Different types of embryonic development probably provoke different paths of BD development. As animals are classified as lower, middle, and higher, the BD of animals could be similarly classified, with the most developed BD existing in humans. Therefore, if NTSs and BDs are the same structure, NTSs may be present on the surfaces of organs in highly developed animals. To our disappointment, NTSs have so far been observed primarily in small animals, such as rabbits [3], rats [4], and mice [10]. Bovine heart is the only large animal organ in which NTSs have been observed [5].

Therefore, we hypothesized that NTS might be easily observable on the surface of swine intestines using trypan blue staining and an optical microscope. 4,6-diamidino-2-phenylindole (DAPI) staining and polarized light microscopy were used to confirm whether any structures obtained fit the definition of NTS. 

## 2. Methods

All experiments were performed in accordance with the Principles of Laboratory Animal Care, prepared by the National Society of Medical Research, and with the Guide for the Care and Use of Laboratory Animals, prepared by the Institute of Laboratory Animal Resources and published by the National Institute of Health (NIH Publication 85-23, Rev. 1985). The study was approved by the Korea University Institutional Animal Care and Use Committee.

Two Yorkshire pigs, weighing 30–40 kg, were used in this study. Preoperatively, the pigs were fasted for 12 h. After intramuscular preanesthetic medication with Rumpun (0.5–1 mg/kg) and Zoletil (7–10 mg/kg), the pigs were placed on an operating table in the supine position with the neck extended. Standard monitoring included electrocardiogram, pulse oximetry, end-tidal CO_2_, and inspiratory and expiratory gas concentration. The auricular vein and femoral artery were cannulated to infuse fluids and for invasive blood pressure monitoring, respectively. On confirming the adequacy of the depth of anesthesia, a laryngeal mask airway (LMA, size 3 for pigs up to 30 kg, size 4 for pigs up to 43 kg) was inserted to the pig and the cuff was inflated with 15 mL of air. Correct LMA placement was confirmed by chest expansion and without leakage of air. Anesthesia was maintained with 50% nitrous oxide in oxygen and an end-tidal concentration of 2-3% enflurane. Intravenous rocuronium bromide was used to facilitate tracheal intubation. After achieving complete muscular relaxation, the LMA was removed and the pig was intubated with a standard cuffed endotracheal tube (Hi-Lo, Mallinckrodt Inc.). The size of the ETT was determined by visual inspection of the larynx and confirmed by the ability to pass the tube without resistance. The pigs received a continuous intravenous infusion of lactated Ringer's solution at 5 mL·kg^−1^·h^−1^ throughout the operation.

The abdominal walls of two swine were carefully dissected along the medial alba. For the tracing experiment with trypan blue, we longitudinally incised the abdominal skin beside the medial alba of each pig. Bleeding from large vessels in the abdominal skin, muscular layers, and peritoneum was controlled using electric cautery. NTSs were identified on organ surfaces under a stereoscopic microscope (SZX12, Olympus, Japan) using small surgical instruments, such as iris scissors, microforceps, and needles, for manipulation. These manipulations were performed carefully because they are the primary step for discriminating NTSs from similar-looking artifacts, such as long, thin pure fibrin coagulations or pieces of peritonea or serosa. In general, NTSs had more regular morphology, elasticity, and color/transparency than artifacts. For NTSs that were difficult to distinguish from artifacts, we used 0.4% trypan blue solution (Sigma, St. Louis, MO, USA) to stain NTSs on the internal organs of swine. After exposure, several milliliters of trypan blue solution were dropped onto the large intestine. About 1 min later, we washed the large intestine several times with phosphate-buffered saline, pH 7.4 (PBS).

The isolated NTS specimens from the large intestine were fixed with either 4% PFA solution or 10% NBF solution for one or two days prior to further analysis and were stored at 4°C. The fixed samples were washed two or three times with PBS solution. To characterize the nuclei in the NTSs, the specimens were stained with 4′,6-diamidino-2-phenylindole (DAPI; Invitrogen, Eugene, OR, USA). A fluorescence microscope (Olympus BX51, Olympus) was used to observe the rod-shaped nuclei. We also confirmed whether phase-contrast images of the NTS samples contained striations using a phase contrast microscope (Olympus BX51, Olympus). 

Finally, we examined the polarizability of the NTS samples using a polarized light microscope (Samwon Optics, Korea) [11]. For this imaging, the NTS sample was placed between two polarizers which are in crossed nicols. Two polarizers were simultaneously rotated for 360° while the position of the sample is fixed, and it was checked for the extinction of polarized light with regard to a constant polarized angle.

## 3. Results

**Figure 1:**
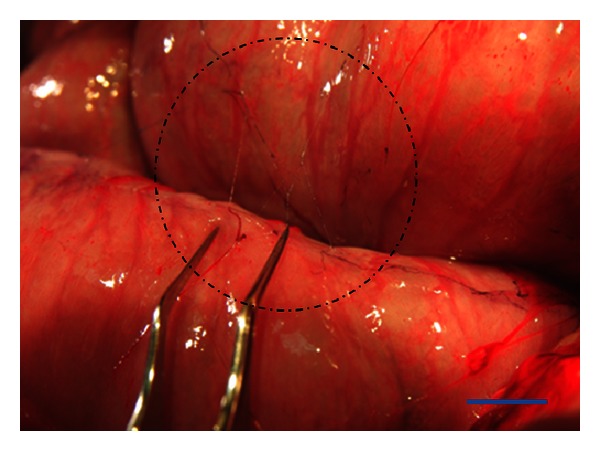
Novel threadlike structures found on the surface of the large intestine of a pig. The circled area indicates the location of the semitransparent threadlike structures that formed a network. Scale bar is 10 mm.

From both pigs, we observed NTSs on the surface of the large intestine, as shown in Figure 1. The elasticity, milky-white color, and semitransparency of the structures were consistent with those reported for small animals, such as rabbits [3], rats [4], and mice [10]. The threadlike structures were uniform in thickness, with a diameter of approximately 30 *μ*m. This diameter was similar to that of NTSs from small animals.

**Figure 2:**
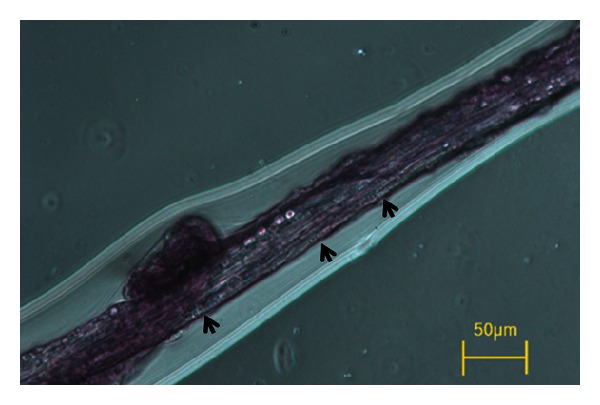
Phase contrast image of a novel threadlike structure from the large intestine of a pig. Three arrows indicate distinctive striations perpendicular to the axis of the vessel.

**Figure 3:**
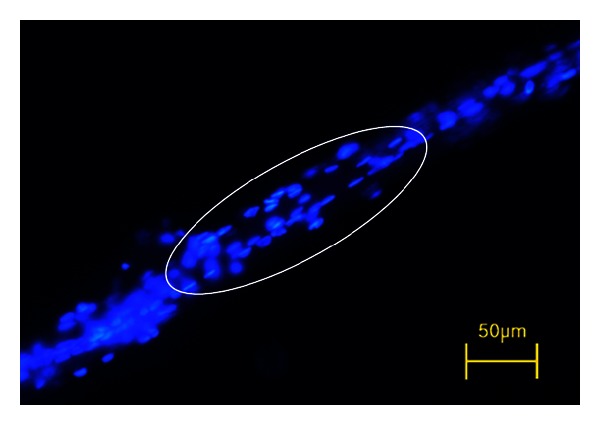
Fluorescence image of DAPI-stained nuclei in a novel threadlike structure (NTS). Rod-shaped nuclei are aligned in a broken-line fashion (dotted-oval circle). This characteristic feature of NTS is commonly seen in other animals and organs.

**Figure 4:**
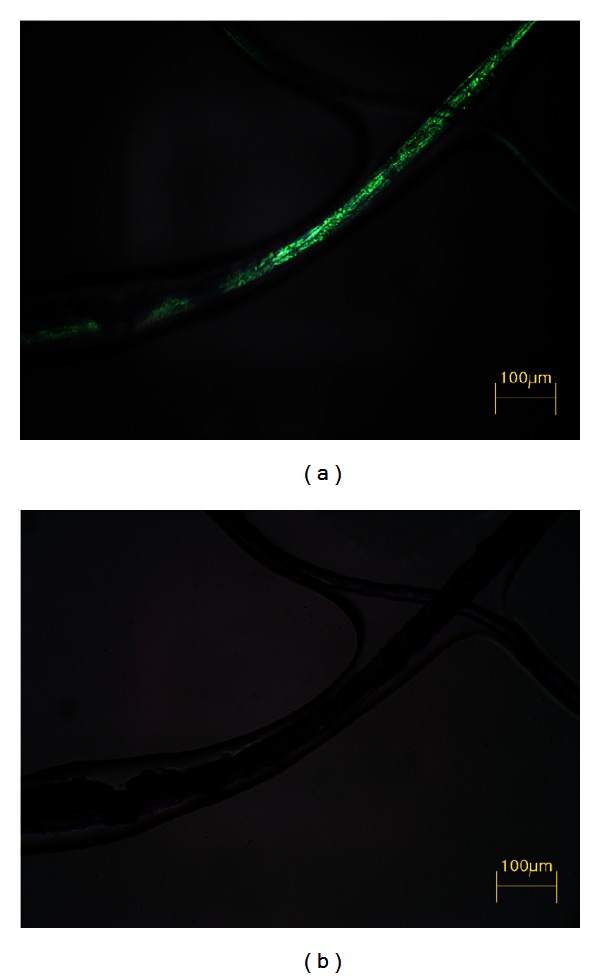
Polarized light microscopic images of a novel threadlike structure obtained from the large intestine of a pig. The NTS appeared to be bright (a), while it becomes dark when the polarized angle is rotated 45° (b). This distinct polarization property means that the NTS contains a significant amount of collagen fibers which are oriented in a single direction. Scale bar is 100 *μ*m.

The phase-contrast microscopy image of the NTSs we obtained had a striped appearance, as shown in Figure 2. This was in agreement with previous primo vessel images. The most striking feature of the NTS samples was the rod shape and linear alignment of the nuclei, as depicted in Figure 3. This shape and alignment of nuclei fulfill the primary necessary condition for the NTSs to be identified as primo vessels, according to Kim [9]. In addition, we obtained polarized light microscopic images, as shown in Figure 4. Using polarized light microscopy, it was confirmed that the intensity of the polarized light was cyclically changed with 90° period. Its maximum intensity and minimum intensity (i.e., extinction) were shown by turns, which is the consistent result with extravascular primo vessel in the brain [11]. 

## 4. Discussion

This is the first report of NTS on the surface of a large animal organ. We found elastic, milky-white, and semitransparent threadlike structures (that formed network structures) *in situ* and *in vivo* on the large intestine of swine. Polarized light microscopy DAPI staining revealed that the observed structures fit the definition of NTS.

Anatomical observations alone indicated that the NTSs were distinctively different from well-known systems like nerves, blood vessels, capillaries, and lymph vessels [8]. The NTSs could be moved freely and could be lifted above the organ surface. There have been no reports of mammalian anatomy in which nerves, blood, or lymph vessels were separated from and raised above the surfaces of organs in normal animals. The NTSs were distinctively different from nerves because liquid flowed out from corpuscles that were sparsely located along the NTS. The NTSs were also easily distinguished from blood capillaries or venules due to erythrocytes because the NTSs were semitransparent and milky white. In addition, the NTSs have been observed inside large arteries and veins [1]. However, the NTSs could be mistaken for lymphatic vessels. It is this possibility of confusion with the lymph system that caused medical and veterinary experts to doubt the existence of NTSs. Indeed, both objects are semitransparent, are of similar size, and carry a transparent liquid. Nevertheless, there are features that clearly distinguish NTSs from lymphatic vessels [4, 12]. The most critical difference between NTSs and lymphatic vessels is that NTSs have subducts, as clearly shown by Shin et al. [12], while lymph vessels and capillary blood vessels have only one lumen, that is, they are single tubular structures. In addition, the presence of 1 ~ 2 mL sized DNA-containing granules in the liquid extracted from the corpuscles is unique to NTSs, whereas lymph vessels carry lymphocytes that are 5 *μ*m or larger. The alignment of rod-shaped nuclei along the major axis of NTSs is accepted as one of the characteristics that can be used to discriminate NTSs from other similar-looking tissues or artifacts [13]. Our fluorescence images of DAPI-stained structures (Figure 3) showed that the rod shape and linear alignment of the nuclei aligned the major axis of the NTS. In addition, the NTS samples showed the same polarization property as the primo vessels in the cranial dura mater (Figure 4). This property is meaningful because it indicates that dense collagen fibers which are oriented in a particular direction exists in the NTS [11]. It also makes the NTSs differentiats from the blood and lymphatic vessels which do not show distinct change of polarized light with regard to the polarization angle. Therefore, we considered the observed network structures on the swine large intestine surface to be NTSs. 

The most striking figure of our result is that thickness of the threadlike structure was uniform, with a diameter of approximately 30 *μ*m. This diameter was similar to that reported for NTS from small animals. Kim primarily used rabbits as laboratory animals. Although Kim did not present data for other animals, he mentioned that NTSs had been observed in various animals, including humans, other mammals, avians, amphibians, fish, and invertebrates, without suggesting any specific animal species [9]. Until now, investigators have tried to confirm the existence of NTSs using the organs of various animals, such as mice, rats, and rabbits. A few case studies have been reported for cows [5] and dogs [14]. As we mentioned previously, as the animals are divided from lower to higher, the NTS of animals can be divided in a similar way. The development of the NTS may be progressive, with the most developed one being in humans. Therefore, it is conceivable that NTSs in large animals might have a larger diameter than the NTSs in small animals because the diameter of blood vessels is relatively large in large animals. According to Lee et al. [5], however, the diameter of NTSs in the bovine heart is 18.38 ± 17.8 *μ*m. Although exact diameter was not presented, NTSs in dogs are estimated to be smaller than 20 *μ*m in diameter [14]. The average NTS diameter in small animals is about 10 *μ*m and ranges from 1 *μ*m to 50 *μ*m [15]. Our results suggest that the diameter of human NTSs may be similar to those of small animals. Such small structures would be difficult to detect with the naked eye. 

There are some limitations to the present study. Histological results of examined specimens should have been presented. However, we were not sure as to whether we would find NTSs in the swine large intestine. Therefore, this study was planned to confirm the presence of NTSs on the intestinal surface of this large animal. The results of the present study provide evidence that NTSs are present on the surface of large-animal organs.

## 5. Conclusion

The authors report evidence that NTSs may be present on surface of large-animal organs, using swine as an experimental model. Our results may make it possible to perform the similar studies using human specimens.
